# Evaluation of the salivary function of patients in treatment with radiotherapy for head and neck cancer submitted to photobiomodulation

**DOI:** 10.4317/medoral.23912

**Published:** 2020-11-28

**Authors:** Lucas Nascimento Ribeiro, Maria Heloisa da Conceição Tavares de Lima, Alessandra Tavares Carvalho, Raylane Farias de Albuquerque, Jair Carneiro Leão, Igor Henrique Morais Silva

**Affiliations:** 1 Resident in oncology dentistry, Department of Dentistry and Prosthetic Rehabilitation, Pernambuco Cancer Hospital - Recife, Brazil; 2 Undegraduate in dentistry, Department of Clinical and Preventive Dentistry, Federal University of Pernambuco - Recife, Brazil; 3 Associate Professor of Oral Medicine, Department of Clinical and Preventive Dentistry, Federal University of Pernambuco - Recife, Brazil; 4 PhD student - Postgraduate Program in Dentistry, Federal University of Pernambuco - Recife, Brazil; 5 Professor of Oral Medicine and Postgraduate Program Coordinator, Department of Clinical and Preventive Dentistry, Federal University of Pernambuco - Recife, Brazil; 6 Coordinator of the Clincal Dentistry Department, Pernambuco Cancer Hospital - Recife, Pernambuco, Brazil

## Abstract

**Background:**

Head and neck radiotherapy is typically associated with toxicities that can have profound effects on the patient's quality of life. Xerostomia, which may or may not be related to hypofunction of the salivary gland, leading to negative consequences, mainly in quality of life, leaving patients more susceptible to the development of oral mucositis, dental caries, oral infection and difficulties in speech is one of the most common side effects of such treatment. The aim of the present study was to evaluate salivary function of patients in treatment with radiotherapy for head and neck cancer submitted to photobiomodulation.

**Material and Methods:**

A cross-sectional study with a quantitative approach was carried out in the Dentistry Department of the Hospital de Câncer de Pernambuco between February and September 2019.

**Results:**

The study sample comprised 23 patients of both genders, treated with radiotherapy for cancer in the head and neck region. The patients were submitted to photobiomodulation with infrared laser, as intraoral applications in order to prevent mucositis and extraoral applications to stimulate salivary glands. The applications were undertaken three times a week on alternate days throughout the radiotherapy period. The following parameters were used: Intraoral 15mW, 12J / cm2, 10s / point, 2.4 J / point, and extraoral 30mW, 7.5J / cm2, 10s / point, 0.3J / point, both with a wavelength of 830nm and area of 0.028cm². Subjective and objective symptoms were evaluated by measuring the unstimulated salivary flow (USF) using the spitting technique before, during and after radiotherapy treatment. For statistical analysis, a significance level of 5% was adopted. Most patients were male (70%) with 60 years of age on average. At the beginning of treatment, 22 patients had USF> 0.2 ml / min (grade 1), at the end of which 15 patients remained unchanged and only 3 patients progressed to grade 3. As for the subjective classification, most (52%) remained in grade 1 (absence of disability) throughout the treatment.

**Conclusions:**

Based upon the results of this study it was possible to conclude that the use of photobiomodulation did not significantly interfere with the xerostomia complaint of patients in treatment with radiotherapy, however, it does seem to prevent patients from reaching higher degrees of xerostomia taking into account salivary flow measures.

** Key words:**Photobiomodulation, head and neck neoplasms, radiotherapy, xerostomia, saliva.

## Introduction

Radiotherapy (RT) and surgery are described as the standard therapies for initial and locally advanced malignant tumors in the head and neck, and may or may not be accompanied by chemotherapy (CT) (1). Despite being one of the most common treatment modalities, RT still produces important acute and long-term side effects in the oral cavity (2). Head and neck RT is typically associated with toxicities that can have profound effects on the patient's quality of life. Among the most common are oral mucositis (OM), dry mouth, dysgeusia, dysphagia, trismus, dermatitis and candidosis (3).

Xerostomia is the most common complaint among patients submitted to isolated treatment with RT or in combination with CT, which may occur during or late in the treatment period (4). Xerostomia is defined as a subjective sensation of dry mouth, which may or may not be related to hypofunction of the salivary glands leading to negative consequences, especially on the quality of life (5). Patients with xerostomia are more susceptible to developing OM, tooth decay, oral infection and speech difficulties (6). As a preventive measure to minimize the effects of xerostomia in these patients, the use of the intensity modulated radiotherapy technique (IMRT) can be mentioned, which has been shown to reduce toxicities in normal tissues, since it irradiates the glandular areas to a lesser extent (7). Other interventions are available, including gland stimulation that may be appropriate for patients with some degree of residual parenchyma of the salivary glands, and can be attempted using sialogogic medications, chewing gums or lozenges. Topical application of salivary substitutes may offer some benefit, providing a moisture retention coating on the oral mucosa. Interventions such as acupuncture have also been used to increase saliva production, possibly increasing peripheral blood flow (8).

A fully effective treatment for RT-induced salivary hypoflow is still not available (9). Since the use of artificial saliva and mechanical and taste stimulants are often not well accepted by patients, and systemic sialogogues can result in important side effects, therapies including the use of low-power laser have been gaining clinical interest in promoting biomodulation of the cellular metabolism, analgesia and anti-inflammatory effects, without mutagenic and photothermal effects (10). However, to date there are few published studies regarding the effectiveness of photobiomodulation (PBM) in preventing xerostomia and salivary hypoflow in patients undergoing cancer treatment.

Due to its low cost and ease of application, lasertherapy is available in the clinical routine of most oncology services, having been used for a long time for the treatment and prevention of mucositis induced by RT and CT (11). In view of the above, it is of utmost importance to carry out studies that evaluate the effectiveness of low-level laser treatment in preventing hyposalivation, as this is a complex condition that has been shown to have negative effects on the quality of life of individuals who need to be submitted to such treatment. The present study aimed to assess changes in the salivary function of patients in treatment with RT for head neck cancer submitted to PBM at the Pernambuco Cancer Hospital (HCP).

## Material and Methods

The study was an analytical cross-sectional with quantitative approach, involving patients in treatment with RT for head neck cancer submitted to extraoral PBM. The non-probabilistic sample consisted of patients with indication for RT treatment for malignant head and neck cancer at the HCP and referred to the Dentistry Department for intraoral PBM. Data collection was carried out from February 2019 to September 2019, and comprised 23 patients. Among the inclusion criteria of the study were the minimum of 18 years of age and diagnosis of malignancy in the head and neck region. In addition, patients should have been treated with RT alone or concomitantly with chemotherapy or adjuvant surgery, the total dose should be equal to or greater than 50Gy and the treatment should include the larger salivary glands, oral cavity or oropharynx. Patients with other possible causal factors of xerostomia/hyposalivation, such as those with diabetes mellitus, autoimmune, infectious and collagen diseases, and patients who used drugs that could interfere with salivary flow (antidepressants, benzodiazepines, anti- hypertensives, among others) were excluded. Patients with indication for palliative radiotherapy, with initial tumors (T1 and T2) and who were unable to answer the questions were also excluded.

All patients underwent oral preparation and adequacy prior to RT, which included periodontal and restorative treatment, extractions, removal of factors that could influence the severity of the acute and late effects of RT (poorly adapted prostheses, inadequate restorations) and suspension advice the use of removable prosthetic appliances. They were also informed about the most frequent oral complications and advised on oral hygiene. All were evaluated three times a week during RT, according to the service routine.

The laser device used was the Flash Laser III (DMC, São Paulo, Brazil) of gallium and aluminum arsenide (AsGaAl), at a wavelength of 808 nm (infrared laser). The following parameters were used, based on studies by Lima *et al*., [2010] and Palma *et al*., [2017]: Intraoral application: 15mW, 12J / cm2, 10s per point, 2.4 J / point (12). Extraoral application: 30mW, 7.5J / cm2, 10s per point, 0.3J / point (13). The type of optical conductor was silica fiber, 10 cm long and an area of 0.028 cm². The photobiomodulation protocol followed was three times a week on alternate days and always by the same dentist. PBM was started before the first RT session and ended after the last RT session. For intraoral application, always excluding the tumor area, three points were illuminated on both jugal mucous membranes (right and left), three on the upper lip and three on the lower, two on the hard palate, one on the soft palate, one on the back of the tongue, two on the edges of the tongue bilaterally, one on the right tonsil pillar and one on the left, and two on the oral floor. For extraoral application, six points were illuminated in each parotid gland and three in each submandibular gland bilaterally. The optical fiber of the laser handpiece was always placed perpendicularly and in contact with the fabric during applications. The chemical disinfection method (70% alcohol) was used to clean the device, in addition to an individual plastic barrier. During treatment, the laser operator and the patient wore goggles with specific lenses.

To determine and classify xerostomia, the Xerostomia Inventory was used according to Thomson *et al*. [1999] in the Portuguese version, which evaluates values from 11 to 55 according to severity (14) and the Table proposed by Eisbruch *et al*., [2003] that evaluates a subjective factor with grades from 1 to 3 and another objective related to the salivary flow rate (not stimulated) also with a grade that varies from 1 to 3 (15). The patients were classified according to the answer through an interview.

Salivary flow and xerostomia classification were recorded in four moments:

D0 - before starting RT treatment

D1 - in the middle of RT treatment (approximately 17th session)

D2 - At the end of RT treatment

D3 - One month after the end of RT treatment

Sialometry with determination of unstimulated salivary flow (USF) was performed by the main researcher. The technique of collecting saliva chosen was "Spitting": patients were instructed to remove any type of oral prosthesis, to be seated in a chair with their head slightly lowered, to swallow the first saliva as soon as asked to start of saliva collection, in disposable cups, for 5 minutes. For the collection, a precision scale was used, where the saliva deposit containers were weighed before the beginning and after the saliva collection. Values above 0.25 ml/min of USF were considered normal. To calculate the total salivary flow, and assuming that 1 g of saliva corresponds to 1 ml, the conversion formula was used (16):

Salivary flow (ml/ min) = Weight of tube after (g) – Weight of tube before (g)/Time of saliva collection (min)

Statistical Package for the Social Sciences (SPSS 13.0) for Windows and Excel 2010 were used for statistical analysis. All tests were applied with 95% confidence. Table 1 shows the results with their respective absolute and relative frequencies. In the other Tables, the numerical variables are represented by measures of central tendency and measures of dispersion. To verify the existence of an association, Fisher's exact test was used for categorical variables and the Spearman's correlation coefficient. The mixed linear regression model was used, which takes into account the possible correlation between the values of the response variable that constitute repeated measures.

## Results

The sample consisted of 23 patients, with an average of 60 years of age. Males comprised 70% of the study sample (16/23). More than 90% of patients reported presente or past smoking and/or alcohol comsuption. Among occupations, most of the sample consisted of retirees or agricultural workers. The most common type of malignancy was oral squamous cell carcinoma, 35% of the tumors were located in the oropharynx and 30% in the larynx. Most tumors had stage IV (44%). Among the types of RT, three-dimensional was the treatment modality that most patients underwent (91%). When the treatments performed were analyzed, concomitant CT + RT was responsible for 48% of the group and cisplatin was the drug of choice in all cases. Surgery was performed in 48% of the group, among the operated tumors 3 were located on the tongue, 2 on oropharynx and 6 laryngectomies were undertaken. Among the complications presented during RT, the most prevalent was xerostomia, which affected 74% of patients, followed by dysphagia, which affected 70% of patients and the prevalence of OM was 57% (Table 1).

**Table 1:**
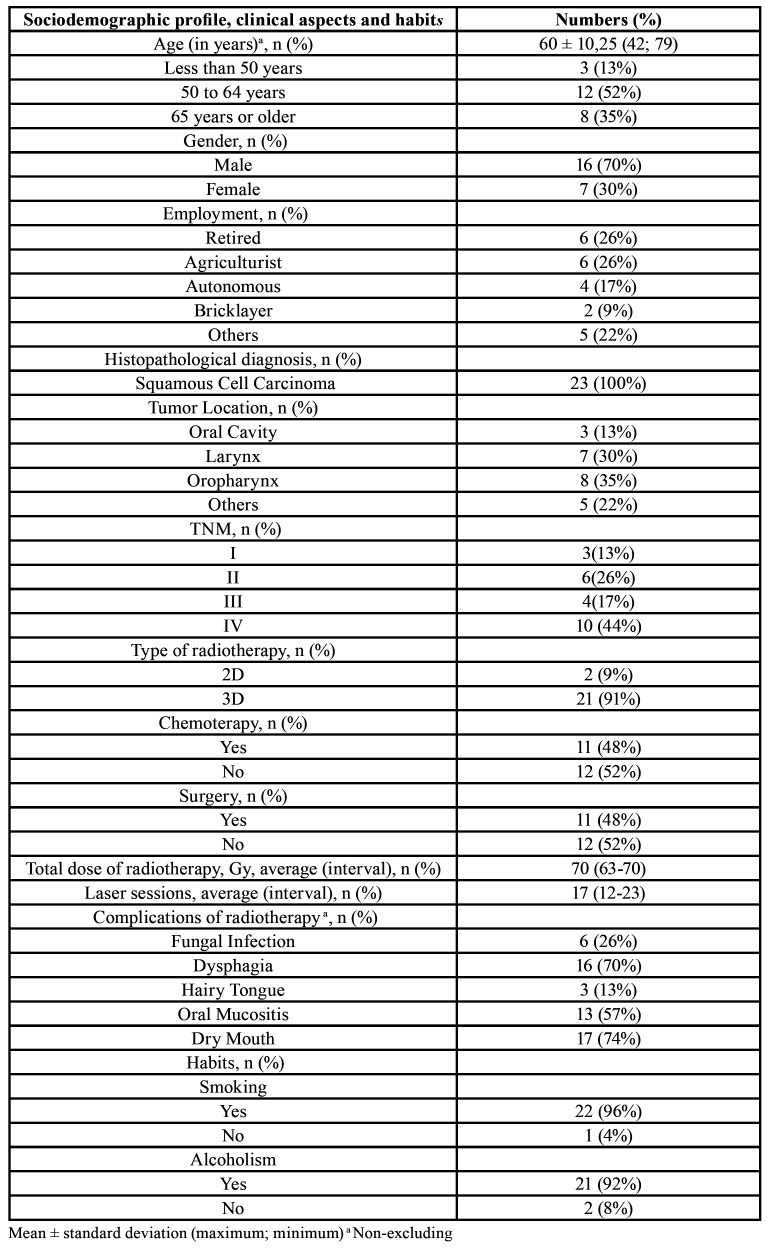
Sociodemographic profile, clinical aspects and habits of patients diagnosed with malignant lesions in the head and neck region treated at the Pernambuco Cancer Hospital.

Table 2 lists the classification of xerostomia, performed by the Xerostomia Inventory, and the time of treatment. It shows that there was a statistically significant difference in the variables "subjective symptoms" and "objective symptoms" in relation to different periods. In the analysis of subjective symptoms, it is shown that 87% of the patients had Grade 1 before RT and after treatment, a minority (17.4%) worsened, progressing to grade 3. As for objective symptoms, it is observed that 95.7% of the patients had Grade 1 before RT and after the end 47.8% of the patients remained in Grade 1, thus presenting an unstimulated salivary flow within the normal range.

When evaluating values of salivary volume and xerostomia inventory, it is observed that there was only a statistically significant difference in the variable “Inventory” in relation to the period analyzed (Table 3). The average salivary volume at before radiotherapy was 0.53 ml / min, being reduced to 0.42 ml / min at the end of the treatment and 0.30 ml / min one month after later. Despite the gradual reduction over the period, the mean at the end of treatment does not characterize the salivary flow rate as low. The xerostomia inventory showed an average response of 14 at the beginning of treatment, remaining constant during and at the end with an average value of 23 with a slight reduction one month after the end of radiotherapy. Important to note thar that the analysis varies between 11 and 55 points.

When the USF and the xerostomia severity were related to the RT dose, there was only a statistically significant correlation between RT dose (Gy) and the salivary volume at the time D0 (*p* = 0.027). However, there is no clinical significance, since at the time of D0 the patient was not yet exposed to salivary changes resulting from RT. The values of the Spearman coefficient also show that there was no correlation between the variables, since the changes in the salivary volume and in the inventory values do not proportionally follow the changes in the RT dose (Gy) (Table 4).

**Table 2:**
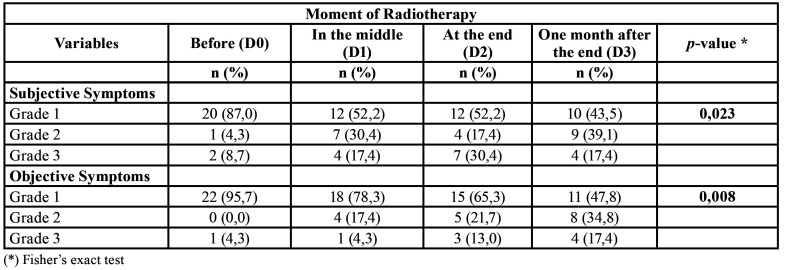
Relationship between xerostomia classification and the time of treatment (radiotherapy).

**Table 3:**
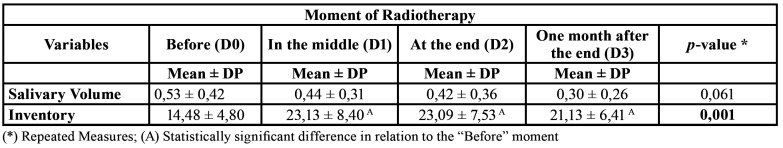
Analysis of the variables salivary volume (ml / min) and xerostomia inventory in relation to the time of treatment.

**Table 4:**
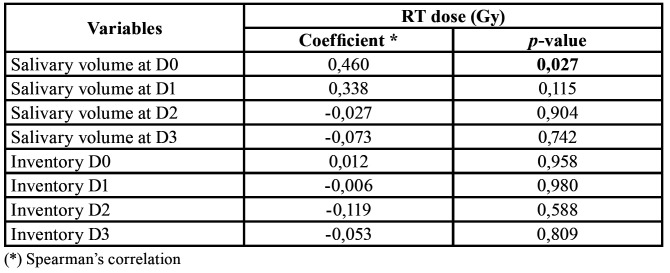
Relationship between the variables salivary volume, xerostomia inventory and radiation.

## Discussion

The present study more than 90% of patients had a history of smoking associated with alcohol comsuption during their lifetime, data that corroborate with studies that bring use of alcohol and smoking as the main agents acting in the pathogenesis of head and neck malignancy (17).

Due to the anatomical location of malignant tumours of the head and neck and the relatively high radiosensitivity of the tumors, RT is among the treatments of choice to achieve a satisfactory prognosis. For advanced stage tumors, RT in combination with CT and / or surgery is the standard treatment, aimed at local and metastatic control (10,18). Our findings corroborate what the literature presents regarding the indicated treatments, since part of the sample was submitted to concomitant RT + CT. Surgery was not commonly used in this subset of patients and can be justified by the advanced staging and the predominance of anatomical locations found in the sample.. The literature points out that in the last twenty years, the standard treatment for RT of the head and neck squamous cell carcinoma has gone from RT 2D to RT 3D and later to RT with modulated intensity (IMRT) (19). In our sample, treatment with RT 3D was predominant, followed by RT 2D. None of our patients underwent IMRT once this technology is still not available in the hospital where the study was conducted.

In the absence of the most modern techniques, RT in the head and neck continues to be associated with toxicities that may be acute or delayed to the detriment of irradiation of adjacent normal tissues (18,20). Among the most common complications are OM, xerostomia, dysgeusia, dysphagia, trismus, dermatitis and candidiasis, which goes against our findings where the most frequent complications were xerostomia, dysphagia, OM and fungal infection (3). Patients who presented fungal infection were diagnosed based upon clinical and symptomatological criteria. Such oral complications were already expected taking into account the fact that the patients were submitted to radiotherapy techniques that do not allow the greater preservation of normal tissues surrounding the tumor. OM was also very frequent complication and was classified according to the parameters of the World Health Organization, in these cases PBM acted in the treatment of OM.

The literature highlights that the occurrence of hyposalivation is related to several factors, such as the radiation dose, the volume of irradiated tissue and the use of concomitant CT for radiation sensitization (21). Regarding the relationship between the RT dose and the dysfunction of the salivary glands, in our sample, all patients were exposed to the dose that could be irreversibly damaged, since the doses varied between 63Gy and 70Gy. The literature shows that the damage to the salivary glands becomes irreversible after cumulative doses greater than 50Gy (8). We believe that because the entire sample was exposed to sufficiently high doses, it was not possible to establish comparative parameters between the RT dose, salivary volume and xerostomia inventory, due to the small sample and the absence of large dose variations between the sample.

Hyposalivation induced by RT is often associated with secondary complications such as radiation decay, dysgeusia, dysphagia, odynophagia, difficulty sleeping and speaking that significantly affect the quality of life of patients (8). These complications influence the appetite and intake of these patients, which can lead to inadequate levels of energy and nutrients, causing malnutrition. One study showed that dry mouth was the most important factor associated with weight loss in cancer survivors after completion of RT (22). This malnutrition can lead to interruption in treatment with impaired prognosis, as well as a negative impact on the quality of life of these patients. In our sample, since all remained with USF as expected, no patient had to interrupt treatment for oral complications, corroborating what has been described in the literature.

To date, there are few data available on the effectiveness of extra oral PBM for preventing xerostomia/hyposalivation in patients undergoing cancer treatment. Recent studies have shown that low-intensity, low-level laser therapy was able to stimulate salivary glands and increase the total protein concentration in the parotid glands of rats ratos (23,24). Other studies have shown that patients undergoing PBM to treat OM reported an improvement in saliva production and the ability to swallow (25). Our results demonstrated that the protocol used with infrared laser was able to keep patients with USF within normal parameters during and after RT. Our results demonstrated that the two protocols used with infrared laser were able to keep patients with USF within normal parameters during and after RT. On the other hand, it should be noted that one month after the end of RT (D3), the salivary volume of patients decreased. This finding demonstrates that the stimulus performed by extra oral PBM was able to maintain increased flow during treatment, but did not prevent the post-termination reduction in RT.

When evaluating the average of laser sessions performed, it is observed a balance in relation to the patients in the sample, which allows to understand that the average was close to 3 weekly sessions, a number stipulated in the methodology. These data corroborate the findings of another study where, a group that received irradiation once a week with another that received three times a week, showed that patients undergoing extra oral PBM three times a week did not show a significant reduction in flow salivate compared to the other group (24). On the other hand, a study reported that in the parameters used, low-level laser therapy was not able to increase USF or decrease xerostomia and associated these negative results with the late effects of RT on the glandular structure, such as fibrosis and acinar atrophy (26).

Our limitations, similar to the studies already published, are the difficulty in obtaining a satisfactory sample and homogenizing the groups due to the profile of the patients and the treatments indicated.

The results of this study suggest that the use of PBM did not prevent the reduction of salivary flow associated with RT, but it did appear to prevent patients from progressing to higher degrees when measuring salivary flow, although further studies are needed, especially randomized and controlled clinical trials in order to confirm that PBM can be used to prevent xerostomia and salivary hypoflow in patients undergoing cancer treatment, in an attempt to improve quality of life.
